# Sexually Transmitted Infections among HIV-1-Discordant Couples

**DOI:** 10.1371/journal.pone.0008276

**Published:** 2009-12-14

**Authors:** Brandon L. Guthrie, James N. Kiarie, Susan Morrison, Grace C. John-Stewart, John Kinuthia, William L. H. Whittington, Carey Farquhar

**Affiliations:** 1 Department of Epidemiology, University of Washington, Seattle, Washington, United States of America; 2 Department of Medicine, University of Washington, Seattle, Washington, United States of America; 3 Department of Global Health, University of Washington, Seattle, Washington, United States of America; 4 Department of Obstetrics and Gynaecology, University of Nairobi, Nairobi, Kenya; Université de Toulouse, France

## Abstract

**Introduction:**

More new HIV-1 infections occur within stable HIV-1-discordant couples than in any other group in Africa, and sexually transmitted infections (STIs) may increase transmission risk among discordant couples, accounting for a large proportion of new HIV-1 infections. Understanding correlates of STIs among discordant couples will aid in optimizing interventions to prevent HIV-1 transmission in these couples.

**Methods:**

HIV-1-discordant couples in which HIV-1-infected partners were HSV-2-seropositive were tested for syphilis, chlamydia, gonorrhea, and trichomoniasis, and HIV-1-uninfected partners were tested for HSV-2. We assessed sociodemographic, behavioral, and biological correlates of a current STI.

**Results:**

Of 416 couples enrolled, 16% were affected by a treatable STI, and among these both partners were infected in 17% of couples. A treatable STI was found in 46 (11%) females and 30 (7%) males. The most prevalent infections were trichomoniasis (5.9%) and syphilis (2.6%). Participants were 5.9-fold more likely to have an STI if their partner had an STI (P<0.01), and STIs were more common among those reporting any unprotected sex (OR = 2.43; P<0.01) and those with low education (OR = 3.00; P<0.01). Among HIV-1-uninfected participants with an HSV-2-seropositive partner, females were significantly more likely to be HSV-2-seropositive than males (78% versus 50%, P<0.01).

**Conclusions:**

Treatable STIs were common among HIV-1-discordant couples and the majority of couples affected by an STI were discordant for the STI, with relatively high HSV-2 discordance. Awareness of STI correlates and treatment of both partners may reduce HIV-1 transmission.

**Trial Registration:**

ClinicalTrials.gov NCT00194519

## Introduction

While access to antiretroviral (ARV) medication will greatly improve outcomes for people living with AIDS in sub-Saharan Africa and may reduce transmission to susceptible partners [1], [2], these drugs alone may not stem the tide of new infections [3]–[5]. In sub-Saharan Africa, an estimated 22.5 million people are living with HIV-1 infection [6], the majority of whom are in stable relationships [7]. In many areas with mature AIDS epidemics, up to 15% of all couples are HIV-1-discordant and the majority of new heterosexually acquired HIV-1 infections occur within such discordant couples [8]–[11]. Interventions that appreciably reduce the risk of transmission within HIV-1-discordant couples could prevent a large proportion of all new infections [11], and therefore these couples are an important population on which to focus prevention efforts [12], [13].

The presence of other sexually transmitted infections (STIs), particularly ulcerative infections, is an important correlate of HIV-1 transmission within discordant heterosexual couples [14]–[21]. While the risk of HIV-1 transmission within discordant couples is relatively low during the chronic phase [13], [22], periods of elevated viral load and the presence of other STIs in one or both partners are associated with significantly increased transmission risk [14], [17], [23], [24]. Because treatment of STIs often lowers genital shedding of HIV-1 [19], [25], [26], timely diagnosis and treatment of STIs, as part of a package of best prevention practices, may be an important component in controlling the risk of HIV-1 transmission in discordant couples.

Three community interventions trials have investigated the effect of enhanced STI treatment on the population-level incidence of HIV-1 infection [27]–[29], only one of which found a benefit associated with improved STI case management [27]. These discrepant results have been attributed to differences in the design of the interventions, as well as differences in the stage of the HIV-1 epidemics, background prevalence of STIs, and existing STI treatment services [30]. There is more consistent evidence that couple-level STI interventions that combine treatment and behavioral counseling can reduce STI prevalence, risky sexual behavior, and HIV-1 transmission [27], [29], [30].

Given the risk posed by the presence of an STI in either partner in an HIV-1-discordant couple, addressing the couple as a unit rather than as separate individuals may result in more effective treatment of STIs and lower risk of HIV-1 transmission. We examined the prevalence and correlates of STIs in a cohort of Kenyan HIV-1-discordant couples and investigated the relationship between the presence of an STI in one partner and the likelihood of an STI in the other partner.

## Methods

### Study Participants

This study was conducted within a randomized clinical trial testing the effectiveness of suppressive acyclovir therapy to prevent HIV-1 transmission among stable HIV-1-discordant couples where the HIV-1-infected participant was HSV-2-seropositive (the Partners in Prevention HSV/HIV Transmission Study, which operated at 14 African sites, including Nairobi) [8]. HIV-1-discordant couples were recruited from voluntary counseling and testing (VCT) centers and antenatal clinics in Nairobi, Kenya from 2004–2007. Eligible couples reported sex with each other ≥3 times in the 3 months prior to screening, planned to remain together for the duration of the study, and were able to provide independent informed consent for participation in the study. HIV-1-infected participants were HSV-2-seropositive and had CD4 counts ≥250 cells/µl, did not have a history of clinical AIDS (WHO stage IV), and were not currently on antiretroviral therapy (ARV) or eligible for ARVs by Kenyan national guidelines.

At the enrollment visit, clinical staff administered a questionnaire including sociodemographic, sexual behavior, and medical history characteristics. Included among these questions, participants were asked if they had been diagnosed with a genital infection or if they had any genital sores in the 3 months prior to enrollment. Participants were interviewed individually to ensure confidentiality.

### Identification of STIs and Laboratory Methods

At enrollment, all participants underwent a physical examination and specimens were collected to test for HIV-1, HSV-2, syphilis, *Chlamydia trachomatis*, *Neisseria gonorrhoeae*, *Trichomonas vaginalis*, bacterial vaginosis (BV), and vaginal candidiasis. HIV-1 testing was by 2 different rapid tests conducted in parallel using a combination of Uni-Gold Recombigen (Trinity Biotech plc, Wicklow, Ireland), Determine HIV 1/2 (Abbott Laboratories, Abbott Park, IL), and Vironostika HIV-1 Microelisa (bioMérieux Inc., Durham, NC), with confirmation by ELISA. Testing for HSV-2 was by Focus ELISA (Cyprus, CA), using a cutoff of 3.5 to improve specificity [31]. HIV-1-infected participants with indeterminate results (1.1–3.4) were eligible if confirmed by Western Blot [32]. Testing for syphilis was by rapid plasma reagin (RPR) test (Becton and Dickinson, Franklin Lakes, NJ) with confirmation by *T. pallidum* haemagglutination assay (TPHA) (Randox Laboratories Ltd, Crumlin, UK). Testing for *C. trachomatis*, *N. gonorrhoeae*, and *T. vaginalis* was conducted on urine samples for males and cervical swabs for females. Testing for *C. trachomatis* and *N. gonorrhoeae* was by the Gen-Probe Combo 2 assay following manufacturer's protocol (Gen-Probe, San Diego, CA). Testing for *T. vaginalis* used TV analyte-specific reagents with APTIMA General Purpose Reagents (Gen-Probe, San Diego, CA). TV analyte-specific reagents include target capture, transcription-mediated amplification (TMA), and hybridization protection, using primers and probes that target TV rRNA [33]. Specimens consistently yielding relative light unit (RLU) values >100,000 were considered positive. BV was defined by a Nugent score of 7–10 on a Gram stain smear. Vaginal candidiasis testing was by wet preparation. PAP smear slides were read using the Bethesda system and findings of cervical intraepithelial neoplasia (CIN) grade I or greater were classified as abnormal cervical cytology. All participants with positive STI results or abnormal cytology were treated per Kenyan national guidelines.

### Statistical Methods

Prevalence of an STI was calculated for each etiology by participant gender and HIV-1 status. Additionally, the prevalence of any treatable STI (syphilis, gonorrhea, chlamydia, or trichomoniasis) was calculated. A couple was classified as affected by an STI if one or both partners had an STI. In the event that STI results were missing for one partner, the couple was classified as affected if the other partner had an STI; however, if the other partner was STI negative, couple STI status was classified as unknown.

The prevalence of STIs was compared between HIV-1-infected and uninfected participants, stratified by gender, using an odds ratio (OR) with a p-value based on Fisher's exact test. Individual-level and couple-level characteristics were accessed as correlates of a prevalent STI. Dichotomous characteristics were compared between those with and without a current STI using a χ2 test (Fisher's exact test was used for infrequent events), while continuous and non-dichotomous categorical characteristics were assessed using logistic regression. In assessing correlates of a prevalent STI, adjustment for multiple comparisons was accomplished by controlling the false discovery rate at 5% and presenting corrected p-values [34]. Tests for a difference in ORs between groups were conducted by including an interaction term in a logistic regression model. All analyses were conducted using STATA statistical software (STATA Corp., College Station, TX).

### Ethics Statement

Human experimentation guidelines of the US Department of Health and Human Services were followed and approval for the study, including forms for written informed consent, was obtained from the Institutional Review Board of the University of Washington and the Ethics and Research Committee of the Kenyatta National Hospital. Written informed consent was obtain from all study participants. The study was conducted following Good Clinical Practice (GCP) and Good Clinical Laboratory Practice (GCLP).

## Results

### Demographic Characteristics

A total of 416 HIV-1-discordant couples were enrolled, of which 322 (77.4%) couples had a female HIV-1-infected partner (Table 1). The median relationship length was 5.2 years (IQR 2.5–10). Females (median age 30 years) were significantly younger than males (median age 36 years; P<0.01, Wilcoxon rank-sum test). Most (94.7%) couples reported that they were married.

**Table 1:** Characteristics of study participants.*

Characteristic	Female (N = 416)		Male (N = 416)	
	Median (IQR†) or Number (%)			
**Demographic**				
**HIV-1 infected**	322	(77.4)	94	(22.6)
**Age**	30	(26, 34)	36	(30, 41)
**Married**	395	(95.0)	396	(95.2)
**Monthly income (US dollars)** ‡	66.71	(40.03, 133.42)	93.40	(53.37, 200.13)
**Education (years)**	9	(7, 12)	11	(8, 13)
**Employed**	155	(37.7)	364	(88.6)
**Reproductive History**				
**Prior pregnancies**	2	(2, 3)	-	-
**Living children**	2	(1, 3)	2	(1, 3)
**Circumcised**	-	-	345	(82.9)
**Current oral contraceptive use**	15	(3.6)	-	-
**Sexual History**				
**Age of first intercourse**	18	(16, 19)	17	(15, 19)
**Lifetime sexual partners**	3	(2, 4)	5	(3, 10)
**Any genital infection in past 3 months** §	72	(17.3)	30	(7.2)
**Genital sores in past 3 months**	80	(19.2)	61	(14.7)
**Sex with someone else in past month**	4	(1.0)	5	(1.2)

* Numbers may not add to total because of missing data.

† IQR, interquartile range.

‡ Based on conversion rate of 1 USD  =  74.95 Kenyan Shillings.

§ Includes urethritis, GUD, LGV, vaginitis, cervicitis, PID, syphilis, gonorrhea, chlamydia, trichomoniasis.

### Sexual Behavior

Median age at first sexual intercourse was 18 years among men and 17 years among women, and women reported a median of 3 lifetime sexual partners compared to 5 among men (P<0.01, Wilcoxon rank-sum test). Couples reported a median of 4 sex acts per month (IQR: 3–8), with no significant difference in the number of acts reported by the male and female partners (P = 0.63, T-test). The majority of participants (79.1% of women and 77.0% of men) reported no unprotected sex acts with their study partner in the past month. Sex with someone other than the study partner in the past month was reported by 4 (1.0%) women and 5 (1.2%) men, and in all cases these participants were HIV-1-infected.

### Prevalence of Genital Infections in Participants

The most prevalent treatable STIs among all participants were trichomoniasis and syphilis (Table 2). The prevalence of trichomoniasis ranged from 2.1% to 8.5% with the highest prevalence among HIV-1-infected females and the prevalence of syphilis ranged from 1.9% to 3.2% with the highest prevalence among HIV-1-uninfected females and HIV-1-infected males. A treatable STI was identified in 46 (11.4%) women and 30 (7.2%) men. Prevalence of a treatable STI was not different comparing HIV-infected and uninfected participants, stratified by gender (P>0.30). Bacterial vaginosis was common, with 24 (29.6%) HIV-1-uninfected women and 89 (32.0%) HIV-1-infected women testing positive for BV (OR = 1.12; 95% CI 0.63–2.01; P = 0.68). Abnormal cervical cytology was observed in only 1 (1.2%) HIV-1-uninfected woman compared to 17 (5.6%) HIV-1-infected women (OR = 4.97; 95% CI 0.75–210.06; P = 0.14).

**Table 2:** Prevalence of genital infections.*

	Female					
	HIV-1 (−) (N = 94)		HIV-1 (+) (N = 322)			
**Characteristic**	N	%	n	%	OR	95% CI
**Any treatable STI** †	7	(8.1)	39	(12.3)	1.58	(0.67, 4.35)
**Genital ulcer disease**	1	(1.1)	5	(1.6)	1.39	(0.15, 66.38)
**Syphilis**	3	(3.2)	10	(3.1)	0.97	(0.24, 5.61)
**Gonorrhea**	0	(0.0)	0	(0.0)	NA	NA
**Chlamydia**	1	(1.2)	2	(0.6)	0.53	(0.03, 31.83)
**Trichomoniasis**	3	(3.5)	27	(8.5)	2.54	(0.75, 13.41)
**Bacterial vaginosis**	24	(29.6)	89	(32.0)	1.12	(0.63, 2.01)
**Candidiasis**	1	(1.2)	5	(1.6)	1.35	(0.15, 64.82)
**Abnormal cervical cytology** ‡	1	(1.2)	17	(5.6)	4.97	(0.75, 210.06)

	Male					
	HIV-1 (−) (N = 322)		HIV-1(+) (N = 94)			
	n	%	n	%	OR	95%CI
**Any treatable STI** †	25	(7.8)	5	(5.3)	0.67	(0.19, 1.84)
**Genital ulcer disease**	3	(0.9)	3	(3.2)	3.51	(0.46, 26.52)
**Syphilis**	6	(1.9)	3	(3.2)	1.74	(0.28, 8.31)
**Gonorrhea**	0	(0.0)	0	(0.0)	NA	NA
**Chlamydia**	4	(1.2)	0	(0.0)	0	(0.00, 3.28)
**Trichomoniasis**	16	(5.0)	2	(2.1)	0.47	(0.05, 1.82)

* Numbers may not add to total because of missing data.

† Positive laboratory result for syphilis, gonorrhea, chlamydia, or trichomoniasis.

‡ CIN 1 or greater.

### STI Concordance within Couples

**Figure 1:**
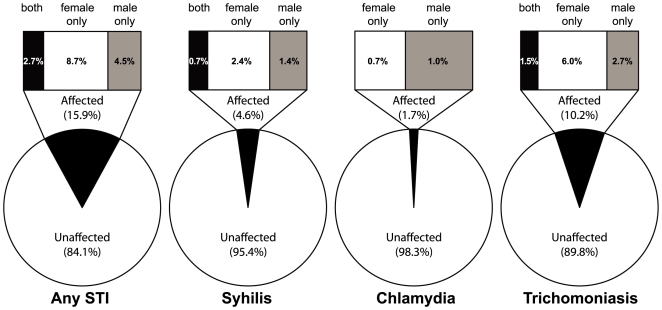
Concordance of treatable STIs identified in female and male partners. Slices of the pie represent the proportion of couples affected and unaffected by an STI. The sections of the upper bar represent the proportion of affected couples in which both partners were infected with an STI, only the female was infected, and only the male was infected.

A treatable STI was found in one or both partners in 65 of 403 (16.1%) couples. Of the 64 couples affected by a treatable STI where the STI status was known for both partners, an STI was identified in both partners in 11 (17.2%) couples, while in 35 (54.7%) couples only the female partner had an STI, and in 18 (28.1%) couples only the male had an STI (Figure 1).

### HSV-2

By study design, all HIV-1-infected participants were HSV-2-seropositive, while 234 (56.3%) HIV-1-uninfected participants were HSV-2-seropositive. Among the HIV-1-uninfected participants 77.7% of women were HSV-2 seropositive compared to 50.0% of men (RR = 1.55; 95% CI 1.33–1.81; P<0.01). Seroprevalence of HSV-2 among the HIV-1-uninfected increased with age among both females (OR = 1.10 per year; 95% CI 1.02–1.18; P = 0.01) and males (OR = 1.06 per year; 95% CI 1.03–1.09; P<0.01). After controlling for age and gender, there was no evidence of an association between HSV-2 seroprevalence and relationship duration (OR = 1.01; 95% CI 0.97–1.06; P = 0.61) or CD4 count of the HIV-1-infected partner (OR = 0.96; 95% CI 0.89–1.04; P = 0.37).

### Correlates of STIs in Men and Women

Among the women, the presence of BV was strongly associated with having a treatable STI (OR = 3.68; 95% CI 1.76–7.79; corrected P<0.01) (Table 3). While not significant after correcting for multiple comparisons, there was a trend indicating that having had genital sores within the past 3 months was associated with a lower likelihood of having a treatable STI (OR = 0.28; 95% CI 0.05–0.90; corrected P = 0.15), with the association restricted to HIV-1-infected participants (OR = 0.17; 95% CI 0.02–0.69; corrected P = 0.03). Among males, we found no significant associations with the presence of a treatable STI, after correcting for multiple comparisons. We also found no significant association between male circumcision and STI prevalence in men (OR = 3.08; 95% CI 0.74–27.21; corrected P>0.90) or women (OR = 1.10; 95% CI 0.46–3.06; corrected P>0.15).

**Table 3:** Correlates of current sexually transmitted infection,* among female and male participants.

Characteristic	Female			Male		
	STI (−) (N = 357)	STI (+) (N = 46)	OR (95% CI)	STI (−) (N = 385)	STI (+) (N = 30)	OR (95% CI)
	n (%)	n (%)		n (%)	n (%)	
**Socio-demographic**						
**Age ≥30 years**	182 (51.7)	19 (41.3)	0.66 (0.33, 1.28)	296 (77.9)	23 (76.7)	0.93 (0.37, 2.66)
**Home with 1 room**	141 (40.1)	13 (28.3)	0.59 (0.28, 1.20)	156 (40.9)	9 (30.0)	0.62 (0.24, 1.45)
**Employed**	131 (37.2)	18 (39.1)	1.08 (0.54, 2.12)	338 (88.9)	25 (83.3)	0.62 (0.22, 2.19)
**Behavioral**						
**First sex <15 years**	27 (7.7)	7 (15.9)	2.26 (0.77, 5.79)	52 (13.7)	9 (30.0)	2.70 (1.03, 6.53)
**Lifetime sex partners**						
1–3	121 (35.0)	11 (23.9)	1 (reference)	48 (12.6)	2 (6.7)	1 (reference)
4–6	178 (51.5)	25 (54.4)	1.54 (0.73, 3.26)	158 (41.5)	11 (36.7)	1.67 (0.36, 7.80)
≥7	47 (13.6)	10 (21.7)	2.34 (0.93, 5.87)	175 (45.9)	17 (56.7)	2.33 (0.52, 10.44)
**Unprotected sex acts** †						
0	279 (80.2)	31 (68.9)	1 (reference)	294 (78.0)	19 (63.3)	1 (reference)
1–2	27 (7.8)	4 (8.9)	1.33 (0.44, 4.06)	34 (9.0)	4 (13.3)	1.82 (0.59, 5.66)
3–10	30 (8.6)	7 (15.6)	2.10 (0.85, 5.18)	37 (9.8)	5 (16.7)	2.09 (0.74, 5.93)
≥11	12 (3.5)	3 (6.7)	2.25 (0.60, 8.41)	12 (3.2)	2 (6.7)	2.58 (0.54, 12.36)
**Vaginal drying used**	208 (58.3)	33 (71.7)	1.82 (0.89, 3.89)	231 (60.0)	17 (56.7)	0.87 (0.39, 2.01)
**Biological**						
**HSV-2-seropositive**	61 (77.2)	6 (85.7)	1.77 (0.19, 85.89)	147 (49.7)	14 (56.0)	1.29 (0.52, 3.25)
**History of STI**	92 (25.9)	7 (15.2)	0.51 (0.19, 1.22)	181 (47.3)	13 (43.3)	0.85 (0.37, 1.93)
**BV**	**88 (28.1)**	**23 (59.0)**	**3.68 (1.76, 7.79)**	–	–	–
**Abnormal cervical cytology** ‡	15 (4.4)	3 (7.3)	1.70 (0.30, 6.40)	–	–	–
**Any birth control**	155 (43.4)	26 (56.5)	1.69 (0.87, 3.33)	–	–	–
**Oral contraceptives**	12 (3.4)	3 (6.5)	2.01 (0.35, 7.83)	–	–	–
**Genital sores** ¶	72 (20.2)	3 (6.5)	0.28 (0.05, 0.90)	58 (15.1)	3 (10.0)	0.63 (0.12, 2.14)
**Any genital infection** ¶,§	56 (15.7)	6 (13.0)	0.81 (0.27, 2.04)	28 (7.3)	2 (6.7)	0.91 (0.10, 3.94)
**Male circumcision**	296 (83.1)	38 (84.4)	1.10 (0.46, 3.06)	314 (82.0)	28 (93.3)	3.08 (0.74, 27.21)
**Partner with current STI**	**18 (5.1)**	**11 (23.9)**	**5.90 (2.31, 14.37)**	**35 (9.4)**	**11 (37.9)**	**5.90 (2.31, 14.37)**

**Bold** indicates significant associations controlling the false discovery rate at 5% based on the Benjamini-Hochberg method.

* Positive laboratory result for syphilis, gonorrhea, chlamydia, or trichomoniasis.

† Unprotected sex acts with study partner in the past month.

‡ CIN 1 or greater.

¶ In the past 3 months.

§ Includes urethritis, GUD, LGV, vaginitis, cervicitis, PID, syphilis, gonorrhea, chlamydia, trichomoniasis.

### Correlates of STI in the Couple

The presence of a treatable STI in one partner was strongly associated with the likelihood of an STI in the other partner. A participant was 5.9-fold more likely to have a treatable STI if their partner also had an infection (OR = 5.90; 95% CI 2.31–14.37; corrected P<0.01). The likelihood of a treatable STI in the couple decreased with the duration of the relationship (P<0.01, test for trend) (Table 4). A treatable STI was more common in couples that reported any unprotected sex in the past month (OR = 2.43; 95% CI 1.34–4.37; corrected P<0.01) and in couples where one or both partners had less than a primary school education (OR = 3.00; 95% CI 1.68–5.37; corrected P<0.01). While not significant after correcting for multiple comparisons, there was a trend indicating that a treatable STI was more common in couples where at least one of the partners reported ≥1 outside sexual partner in the past month (OR = 4.36; 95% CI 1.23–15.49; corrected P = 0.09).

**Table 4:** Couple characteristics associated with a couple being affected by a current treatable sexually transmitted infection.*

Characteristic		
	OR	95% CI
**Married**	0.36	(0.13, 1.10)
**Less than primary education** †	**3.00**	**(1.68, 5.37)**
**CD4 count <400**	1.03	(0.56, 1.93)
**Relationship duration**		
≤1	1	reference
1–3	0.46	(0.19, 1.10)
≥4	**0.30**	**(0.14, 0.64)**
**Age difference (per 5 years)**	0.93	(0.74, 1.16)
**Female index partner**	1.62	(0.77, 3.74)
**Male circumcised**	1.30	(0.59, 3.15)
**Any unprotected sex in past month**	**2.43**	**(1.34, 4.37)**
**Other partners in past month**	**4.36**	**(1.23, 15.49)**

**Bold** indicates significant associations controlling the false discovery rate at 5% based on the Benjamini-Hochberg method.

* Positive laboratory result for syphilis, gonorrhea, chlamydia, or trichomoniasis for either or both partners.

† For either or both partners.

In couples that were discordant for the presence of a treatable STI, the partner with the STI was the female partner in 35 (68.6%) of 51 couples, which was a significantly higher proportion than would be expected by chance (P = 0.01, binomial test). HIV-1 status was not associated with which partner in the couple had the STI (RR = 0.91; 95% CI 0.55–1.53; P>0.90). Among STI-discordant couples the women were 4.5 years younger (95% CI 0.4–8.6 years) than couples where both partners had an STI. STI-discordant couples did not differ from STI-concordant-positive couples in terms of marital status, education, CD4 count, relationship duration, male circumcision status, unprotected sex, or outside sexual partners.

## Discussion

In this population of HIV-1-discordant couples in stable relationships, we found that 12% of females and 7% of males were infected with a treatable STI, with the highest prevalence among the HIV-1-infected females. When considered as a couple, approximately 1 out of every 6 couples was affected by a treatable STI. The presence of an STI in an HIV-1-discordant couple may pose increased risk of transmission of HIV-1 within the couple, making diagnosis and treatment of STIs in these couples an important component of reducing transmission. This study indicates that treatable STIs may be common among HIV-1-discordant couples, even if the relationships are considered stable.

The most common treatable STIs were trichomoniasis and syphilis. Bacterial vaginosis was also common among both HIV-1-infected and uninfected women, with 30–32% positive for BV. Chlamydia was found in only 1% of participants, and gonorrhea was not found in any of the participants. Among HIV-1-discordant couples, identification of an STI in one partner is an opportunity to counsel both partners about reducing their risk of HIV-1 transmission. Treatment for ulcerative and non-ulcerative infections is safe, effective, and cheap using readily available medications, but re-infection from an untreated sexual partners is common, making partner treatment important in STI management [35], [36].

As expected, the presence of a treatable STI was associated with riskier sexual behavior (e.g., unprotected sex). Additionally, low education was significantly associated with STI prevalence, a finding that is consistent with previous studies [37]–[39]. Education is likely a proxy for other socioeconomic factors affecting the risk of acquiring an STI, but it may also be directly related to access to condoms and knowledge about their importance and proper use. The finding that among some participants the presence of genital sores in the past 3 months was associated with lower likelihood of a current treatable STI is initially counterintuitive. This association is most likely due to recent syndromic STI treatment in response to genital ulcers, resulting in decreased prevalence of treatable STIs. Awareness of the risk factors and correlates of treatable STIs in HIV-1-discordant couples can aid in the identification of an STI in couples even if neither partner reports any symptoms.

While only 18% of couples affected by a treatable STI were concordant for the infection, the presence of an STI in one partner was highly associated with the likelihood of an STI in the other partner. STI discordance could arise in a couple as a result of one partner clearing the infection while the other remains infected or in couples where the infection has not yet been transmitted within the couple, or it could be an artifact of failure of the assay to detect infection in one partner. In any case, STI discordance was common, and is likely to lead to transmission of the STI to the other partner if prevention and treatment measures are not implemented. In this study, among the STI discordant couples, the partner with the STI was more likely to be the female partner, but no more or less likely to be the HIV-1-infected partner, highlighting the importance of testing both partners in the couple.

Couple-focused voluntary counseling and testing programs can be effective interventions to reduce HIV-1 transmission in couples [40], [41], achieved in part through increased rates of disclosure of HIV status and increased condom use [42]–[46]. Negative consequences of such programs have been minimal [47], although disclosure can be associated with the break-up of some couples [45]. While the emphasis of couple-focused programs has been on disclosure and VCT, more work is needed to investigate the benefit of ongoing couple-focused management of discordant couples that extends past the disclosure stage. Prevention and treatment of STIs would be a key component of such an approach. The effectiveness of partner STI treatment is well established, but conventional partner treatment programs may not take advantage of the opportunity to provide additional counseling and risk-reduction messages targeted specifically at discordant couples. Despite the potential benefits, couple-focused STI management programs must account for gender-specific needs as well as prevention of negative consequences such as domestic violence.

Prevention of HSV-2 transmission may be another strategic objective in discordant couples given the high proportion of couples found to be HSV-2-serodiscordant. Among this population of HIV-1-infected people who were also HSV-2-seropositive, only 56% of their HIV-1-uninfected partners were also HSV-2-seropositive. HIV-1-uninfected men in particular had a relatively low HSV-2 seroprevalence (50%) compared to females (78%), making the suppression of HSV-2 in their partners and the early identification of HSV-2 outbreaks a potentially valuable intervention [48]. Valacyclovir reduces transmission of HSV-2 [49], but suppression of HSV-2 has not been demonstrated to reduce HIV-1 transmission (Celum et al., in preparation) [50]. Previous studies have shown that messages targeted at stable couples to prevent STIs from outside partners are insufficient to prevent HSV-2 transmission, highlighting the importance of messages tailored to HSV-2 prevention [51]. The relatively high rate of HSV-2 discordance particularly among male HIV-1 uninfected partners provides a potential opportunity for intervention.

This study benefited from a relatively large sample size and was nested within a clinical trial that provided a high level of quality assurance. Nevertheless, some limitations exist. We used a relatively high Focus ELISA cutoff of 3.5 to determine HSV-2 seropositivity, and therefore may have missed some true infections, contributing to some of the HSV-2 serodiscordance. Detection of syphilis is complicated by the difficulty of separating current infections from historic treated infections. While the majority of participants positive for syphilis had an RPR titer of at least 1∶8, indicating recent infection, it is possible that some cases of syphilis represent past treated infections (data not shown). Finally, our ability to generalize the findings of this study to discordant couples in general is limited to some extent by the inclusion and exclusion criteria of the randomized trial design. The study population is likely to be somewhat healthier, due to the criteria of a CD4 count ≥250 cells/µl, and more stable than couples in the general population. This selection likely produced a study population with less risky sexual behavior and fewer outside partners than the general population, resulting in a lower prevalence of treatable STIs. Additionally, by design, all HIV-1-infected participants were HSV-2-seropositive; however, this is representative of the majority of HIV-1-infected individuals in Africa [52].

This study provides useful information about the prevalence of treatable STIs among HIV-1-discordant couples and the factors associated with the presence of these infections. While the periods of acute HIV-1 infection and late-stage disease pose the highest per-contact risk of transmission, the intervening period of latent infection is far longer, and is estimated to account for 44–51% of heterosexual transmissions [53]. Therefore, understanding STIs among discordant couples during latent infection is highly relevant to HIV-1 prevention strategies. Approaches to STI management among HIV-1-discordant couples that do not consider the dynamics of such infections within couples may not fully address their contribution to HIV-1 transmission. We have shown that even among stable discordant couples, the prevalence of STIs and genital infections is relatively high, and that STI discordance is common. Therefore, approaching discordant couples as a unit may improve the outcome of STI management, and facilitate other risk reduction messages and interventions.
